# Prognostic Value of a Cardiopulmonary Exercise Testing‐Derived Summed Score in Idiopathic Pulmonary Fibrosis and Connective Tissue Disease‐Associated Interstitial Lung Disease: A Prospective Cohort Study

**DOI:** 10.1002/resp.70193

**Published:** 2025-12-22

**Authors:** Yu‐Lin Tsai, Kai‐Ming Chang, Chung‐Shih Chin, Chiann‐Yi Hsu, Yi‐Hsuan Yu, Yuan‐Yang Cheng, Pin‐Kuei Fu

**Affiliations:** ^1^ Integrated Care Centre of Interstitial Lung Disease Taichung Veterans General Hospital Taichung City Taiwan; ^2^ Division of Cardiopulmonary Rehabilitation, Department of Physical Medicine and Rehabilitation Taichung Veterans General Hospital Taichung City Taiwan; ^3^ Department of Industrial Engineering and Enterprise Information Tunghai University Taichung City Taiwan; ^4^ Department of Chest Medicine Taichung Veterans General Hospital Taichung City Taiwan; ^5^ College of Health Science Central Taiwan University of Science and Technology Taichung City Taiwan; ^6^ Division of Pulmonary and Critical Care Medicine, and Hyperbaric Oxygen Therapy Centre, Department of Chest Medicine Taichung Veterans General Hospital Taichung City Taiwan; ^7^ Department of Post‐Baccalaureate Medicine, College of Medicine National Chung Hsing University Taichung City Taiwan; ^8^ Biostatistics Task Force, Department of Medical Research Taichung Veterans General Hospital Taichung City Taiwan; ^9^ Division of Clinical Research, Department of Medical Research Taichung Veterans General Hospital Taichung City Taiwan

**Keywords:** cardiopulmonary exercise testing (CPET), interstitial lung disease (ILD), mortality prediction, summed score, survival analysis

## Abstract

**Background and Objective:**

Our previous study demonstrated that a summed score derived from six cardiopulmonary exercise testing (CPET) parameters could predict 1‐year mortality in patients with interstitial lung disease (ILD). However, its long‐term prognostic value across different ILD aetiologies remains unclear. This study aimed to assess the predictive performance of CPET‐derived parameters for long‐term outcomes in patients with idiopathic pulmonary fibrosis (IPF) and connective tissue disease‐associated ILD (CTD‐ILD).

**Methods:**

In this prospective cohort study, 210 patients newly diagnosed with ILD between 2018 and 2022 at a tertiary medical centre underwent CPET. A CPET‐derived summed score was evaluated for its association with a composite outcome of all‐cause mortality or lung transplantation. Cox regression and receiver operating characteristic curve analyses were used to examine predictive ability and identify the optimal cutoff value. Kaplan–Meier survival analysis and log‐rank tests compared event‐free survival in IPF and CTD‐ILD patients.

**Results:**

A summed score incorporating five CPET‐derived variables was an independent predictor of the composite outcome. Patients with scores of 2–5 had markedly lower event‐free survival (44.2%) than those with scores of 0–1 (88.3%). The score demonstrated consistent predictive value in both IPF and CTD‐ILD.

**Conclusion:**

The CPET‐derived summed score is a useful prognostic tool for predicting all‐cause mortality or the need for lung transplantation in newly diagnosed ILD patients. It also retains predictive accuracy for long‐term outcomes in both IPF and CTD‐ILD. External validation in other ILD subtypes is warranted.

**Trial Registration:**

ClinicalTrials.gov identifier: NCT06476470

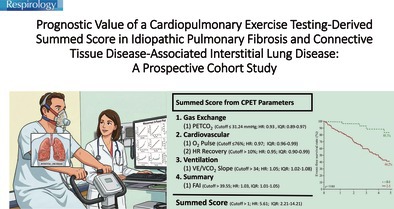

## Graphical abstract

A cardiopulmonary exercise testing‐derived summed score effectively predicted mortality or lung transplantation risk in newly diagnosed interstitial lung disease. Higher summed scores (2–5) were associated with markedly lower event‐free survival rates. This prognostic tool demonstrated consistent predictive value in both idiopathic pulmonary fibrosis and connective tissue disease‐associated interstitial lung disease subtypes.

## 1 Introduction

Interstitial lung disease (ILD) encompasses a heterogeneous group of pulmonary disorders characterised by parenchymal damage, with varied clinical manifestations, imaging features, and prognostic outcomes [1, 2]. Compared with idiopathic pulmonary fibrosis (IPF), connective tissue disease‐associated ILD (CTD‐ILD) involves systemic manifestations affecting multiple organs, including the heart and musculoskeletal structures [3]. Given this complexity, ILD management often requires multidisciplinary discussion (MDD) involving experts from various specialties. Recent studies highlight the pivotal role of MDD in guiding treatment decisions, enabling more individualised and effective therapeutic strategies [4, 5].

The prognosis of ILD varies substantially by subtype. A recent review reported a 5‐year survival rate of 45.6% for IPF [6]. In contrast, 5‐year survival rates for CTD‐ILD range from 40% to 82%, depending on the specific underlying connective tissue disease [7, 8, 9]. Notably, patients with advanced ILD who do not undergo lung transplantation have a median survival of less than 2 years [1]. Given the poor prognosis of ILD, early identification of mortality risk factors is critical for optimising clinical management in newly diagnosed patients. Several established tools, such as the Gender‐Age Physiology (GAP) index, pulmonary function tests, and computed tomography (CT), are widely used to assess disease severity and predict outcomes in ILD [1, 10, 11, 12].

Recent studies have investigated the use of summed scores incorporating clinical parameters and biomarkers—beyond traditional lung function tests—to enhance mortality prediction in patients with ILD [13, 14]. In our previous work, we identified six cardiopulmonary exercise testing (CPET) parameters—peak oxygen consumption ($\overset{`}{V}$O_2_) divided by body weight, oxygen pulse, peak end‐tidal CO_2_ partial pressure (PETCO_2_), heart rate recovery one minute after CPET, the slope of the minute ventilation‐to‐carbon dioxide output ($\overset{`}{V}$E/$\overset{`}{V}$CO_2_), and Functional aerobic impairment (FAI)—as significant predictors of 1‐year mortality in newly diagnosed ILD patients [15]. However, this study was limited by a relatively small sample size (*n* = 106), a short follow‐up period of only 1 year, and the absence of subtype‐specific analysis. Due to patients with IPF generally experience a more rapid decline in lung function and respond poorly to therapy compared to patients with CTD‐ILD [16], a 1‐year follow‐up period may be insufficient to fully evaluate the prognostic utility of CPET‐based risk stratification. To address these limitations, studies with larger cohorts and extended follow‐up are warranted. The study aims to validate the prognostic value of CPET‐derived summed scores in predicting a composite outcome of all‐cause mortality or lung transplantation in ILD patients and to explore its applicability across different ILD subtypes, including IPF and CTD‐ILD.

## 2 Methods

### 2.1 Study Design, Patient Enrolment, and Ethics

The current data is derived from a subgroup analysis of a prospective, single‐centre, real‐world registry study conducted at an ILD referral medical centre in central Taiwan. The Registry of Interstitial Lung Disease (REGILD) has been enrolling newly diagnosed ILD patients since December 28, 2018. Additionally, the REGILD cohort is part of a prospective multicentre ILD registry in Taiwan (NCT06476470). Diagnoses were confirmed through MDD involving pulmonologists, rheumatologists, radiologists, and pathologists. Several studies utilising the REGILD cohort have been published, investigating various prognostic factors [5, 15, 17, 18, 19].

Patients newly diagnosed with ILD between December 28, 2018, and December 31, 2022, were included in the study. Those diagnosed with IPF or CTD‐ILD based on high‐resolution computed tomography (HRCT), clinical and serologic findings, and confirmed by a MDD team were enrolled. All patients underwent CPET during the stable phase of their underlying disease. Patients with contraindications to CPET, as defined by the American College of Sports Medicine (ACSM), were excluded [20]. Individuals with musculoskeletal limitations or active disease affecting exercise performance were also excluded.

Written informed consent was obtained from all participants prior to enrolment. This study was conducted in accordance with the Declaration of Helsinki and was approved by the Ethics Committee of Taichung Veterans General Hospital (IRB number: CE18325B; approval date: December 18, 2018). The study is also registered on ClinicalTrials.gov (NCT06476470).

### 2.2 ILD Assessment Protocol in the REGILD Registry Cohort

Baseline demographics, including age, sex, aetiology of ILD, body mass index, comorbidities, and medication history were collected. Pulmonary function tests and CPET were performed for all participants. The severity of dyspnoea in daily life was assessed using the Modified Medical Research Council (mMRC) questionnaire. The GAP index was used to assess ILD severity. The duration of follow‐up from the date of CPET was also recorded. The comorbidities of enrolled patients were summarised using the Charlson Comorbidity Index (CCI) [21].

### 2.3 Pulmonary Function Test Procedure

As in our previous study [15], forced vital capacity (FVC) and forced expiratory volume in 1 s (FEV_1_) were obtained by spirometry in accordance with the updated technical standards of the American Thoracic Society (ATS) and the European Respiratory Society (ERS) [22]. The diffusion capacity of the lung for carbon monoxide (DLCO) was measured following the 2017 ATS/ERS guidelines for single‐breath carbon monoxide uptake in the lungs [23].

### 2.4 Cardiopulmonary Exercise Testing Procedure

An electromagnetically braked cycle ergometer was utilised, following an incremental stepwise protocol in which pedal resistance increased by 10 watts per minute after a 1‐min rest period and a 3‐min warm‐up period, as previously described [15, 24]. Real‐time measurements of ventilation amount ($\dot{V}E$), $\dot{V}O_{2}$, carbon dioxide production ($\dot{V}{\mathrm{CO}}_{2}$), PETCO_2_, blood pressure, heart rate, oxygen saturation (SpO_2_), and electrocardiography were recorded during symptom‐limited exercise tests. CPET was terminated if patients met specific criteria, including a respiratory exchange ratio > 1.1, the achievement of a $\dot{V}O_{2}$ plateau, or the development of symptoms during exercise that prevented further testing [25]. CPET provides valuable data during exercise testing, with key parameters for patients with ILD including the peak PETCO_2_, O_2_ decrement, peak oxygen pulse (calculated as peak $\dot{V}O_{2}$ divided by heart rate), heart rate recovery 1 min after finishing the incremental phase of CPET, $\dot{V}E$/$\dot{V}{\mathrm{CO}}_{2}$ slope, peak $\dot{V}O_{2}$ divided by body weight, and the peak workload [25, 26]. The predicted $\dot{V}O_{2}$, FAI, maximum voluntary ventilation and breathing reserve were calculated [15, 27, 28].

### 2.5 Outcome Measurement

The primary outcome of this study was all‐cause mortality or lung transplantation. The reference date was defined as the day the participant underwent CPET, and their event status was tracked for 5 years from that date or until the end of the observation period on April 30, 2024, whichever came first. Data on mortality occurrences was obtained from health records maintained by the Taiwan Ministry of Health and Welfare. Subsequent analyses were conducted to assess the predictive value of CPET data for events across different ILD aetiologies. Patients were categorised into the IPF group and CTD‐ILD group to explore whether the predictive significance of CPET‐derived parameters differed between these subgroups.

### 2.6 Statistical Analysis and Acquisition of Summed Score

The demographic data for categorical variables is presented as the number of patients, while continuous parameters are reported as the median with the interquartile range. Categorical variables were analysed using the chi‐squared test or Fisher's exact test, as appropriate. Continuous variables were compared using the Mann–Whitney *U* test. The prognostic value of CPET parameters was assessed using Cox proportional hazards models to assess the time‐to‐event outcome during follow‐up. The proportional hazards assumption was assessed using log‐minus‐log (LML) survival plots. The multivariable analysis was adjusted for age, sex, IPF, and GAP index. Thresholds were derived for parameters with significant prognostic effects using an empirical, censor‐unadjusted receiver operating characteristic (ROC) approach, in which patients alive or censored before 5 years were classified as non‐events, and the optimal cutoff points were determined by Youden's index. A summed score was then calculated by assigning 1 point to each selected parameter that exceeded its threshold. Subsequently, the optimal cutoff value of the summed score was determined using an empirical, censor‐unadjusted ROC approach. Kaplan–Meier estimates and log‐rank tests were applied to calculate and compare event rates during follow‐up. Data analysis was performed using IBM SPSS software version 21.0 and MedCalc software version 22.023. A two‐sided *p* value of < 0.05 was considered statistically significant.

## 3 Results

### 3.1 Baseline Characteristics and Pulmonary Physiology

Between December 28, 2018, and December 31, 2022, a total of 246 patients were newly diagnosed with ILD. After applying the inclusion and exclusion criteria, 210 patients met the eligibility requirements and were enrolled. The median age was 67.5 years (IQR: 60–74), with the majority being female (*n* = 116; 55.2%). The predominant aetiology was CTD‐ILD (*n* = 154; 73.3%). Common coexisting pulmonary diseases included chronic obstructive pulmonary disease (34.1%) and asthma (32.9%), for which all patients received standard treatment. Less common conditions included previous pulmonary tuberculosis, prior nontuberculous mycobacterial infection, pulmonary arterial hypertension, pneumoconiosis, and bronchiectasis.

Based on FVC, DLCO, GAP index and mMRC dyspnoea scores, most patients had mild to moderate disease, with median FVC values: 72% (IQR: 59.0–87.0), DLCO: 67.0% (IQR: 51.0–81.3), GAP index: 3 (IQR: 2–5) and mMRC: 1 (IQR: 0–2). Regarding the CPET data, the median FAI was 37.1% (IQR: 25.7–50.2), indicating a slight reduction in aerobic capacity. At the time of CPET termination, 51.4% of patients exhibited a respiratory exchange ratio (RER) > 1.1. Over a median follow‐up of 2.9 years, the overall event rate was significantly higher in the IPF group (55.4%) compared with the CTD‐ILD group (20.1%).

Compared with patients with CTD‐ILD, those with IPF were older and predominantly male, with higher BMI, greater FEV_1_/FVC ratios, higher GAP index, more frequent use of antifibrotic agents, higher mMRC dyspnoea scores, and a higher incidence of events. In terms of CPET parameters, the IPF group exhibited lower peak PETCO_2_, greater O_2_ decrement, and a steeper VE/VCO_2_ slope. The clinical characteristics and baseline profiles of the study participants are summarised in Table 1.

**TABLE 1:** The demographic characteristics and the initial profiles of the different interstitial lung disease subtypes.

	Total	IPF	CTD‐ILD	*p*
	(*n* = 210)	(*n* = 56)	(*n* = 154)	
Age (years)	67.5 (60–74)	75 (70–80)	65.5 (57–71)	< 0.001**
Sex^a^				< 0.001**
Male	94 (44.8%)	45 (80.4%)	49 (31.8%)	
Female	116 (55.2%)	11 (19.6%)	105 (68.2%)	
Body mass index (kg/m^2^)	23.4 (21.2–26.4)	25.1 (22.2–27.7)	23 (21.1–25.2)	0.006**
Charlson comorbidity index	2 (1–4)	3 (2–4)	2 (1–4)	0.440
Comorbidities				
Cardiovascular diseases^a^	48 (22.9%)	15 (26.8%)	33 (21.4%)	0.414
Cerebrovascular disease^b^	20 (9.5%)	6 (10.7%)	14 (9.1%)	0.723
Pulmonary disease except ILD^a^	167 (79.5%)	47 (83.9%)	120 (77.9%)	0.340
Chronic kidney disease^a^	41 (19.5%)	11 (19.6%)	30 (19.5%)	0.979
Cancer^b^	21 (10.0%)	5 (8.9%)	16 (10.4%)	0.755
Pulmonary function test				
FVC, % predicted	72.0 (59.0–87.0)	71.0 (58.5–88.8)	72.0 (59.0–86.0)	0.988
FEV_1_, % predicted	74.0 (60.5–87.0)	77.0 (60.0–94.0)	73.0 (60.5–86.0)	0.268
FEV_1_/FVC	83.0 (78.0–88.0)	86.0 (78.0–91.0)	82.0 (78.0–86.0)	0.037*
DLCO, % predicted	67.0 (51.0–81.3)	70.0 (53.3–79.8)	67.0 (49.8–83.0)	0.855
GAP index	3 (2–5)	4 (3–6)	3 (1–4)	< 0.001**
mMRC dyspnoea score	1 (0–2)	1 (1–2)	1 (0–2)	0.025*
CPET data				
Gas exchange efficiency				
Peak PETCO_2_, mmHg	30.9 (26.7–34.5)	29.0 (25.1–33.6)	31.4 (27.3–35.1)	0.026*
O_2_ decrement	4 (2–7)	5 (2–9)	4 (2–6)	0.028*
Cardiovascular response				
Oxygen pulse, % predicted	81.5 (64.8–99.0)	80.5 (63.3–98.8)	82.0 (65.0–99.3)	0.913
Heart rate recovery 1 min after CPET, beats/min	9 (5–14)	7.5 (4–13)	9 (5–14)	0.636
Ventilation efficiency				
Breathing reserve, %	48.0 (36.8–58.0)	48.5 (30.0–56.5)	47.5 (38.5–58.3)	0.198
$\dot{V}E$/$\dot{V}{\mathrm{CO}}_{2}$ slope	33.8 (29.6–39.6)	37.0 (32.5–43.4)	32.3 (29.1–37.9)	< 0.001**
Summary				
FAI, %	37.1 (25.7–50.2)	40.6 (19.8–50.2)	35.1 (25.9–49.9)	0.479
Peak workload	50 (40–70)	50 (40–60)	50 (37.5–70)	0.501
Anti‐fibrotic agents during follow‐up^a^	111 (52.9%)	46 (82.1%)	65 (42.2%)	< 0.001**
Lung transplantation	4 (1.9%)	3 (5.4%)	1 (0.6%)	0.059
Event^c^ within follow‐up^a^	62 (29.5%)	31 (55.4%)	31 (20.1%)	< 0.001**
Follow‐up time (years)	2.9 (2.0–4.3)	2.3 (1.5–3.3)	3.4 (2.1–4.9)	< 0.001**

*Note*: Data are presented as median (interquartile range) or *n* (%).

Abbreviations: CPET, cardiopulmonary exercise testing; CTD‐ILD, connective tissue disease‐associated interstitial lung disease; DLCO, diffusing capacity of the lungs for carbon monoxide; FAI, functional aerobic impairment; GAP, gender‐age physiology; IPF, idiopathic pulmonary fibrosis; mMRC, Modified Medical Research Council; PETCO_2_, end‐tidal carbon dioxide partial pressure; $\dot{V}\;E$/$\dot{V}\;{\mathrm{CO}}_{2}$, minute ventilation to carbon dioxide output; $\dot{V}\;O_{2}$, oxygen consumption.

^a^ Chi‐Square test between IPF and CTD‐ILD.

^b^ Fisher's exact test between IPF and CTD‐ILD.

^c^ Event: death or lung transplantation.

* *p* < 0.05.

** *p* < 0.01.

### 3.2 Event Risk Analysis

As shown in Table 2, the study population comprised 148 survivors and 62 participants who either died (*n* = 58) or underwent lung transplantation (*n* = 4). Compared to the survivor group, the event group was older, had a higher proportion of male participants and IPF cases, lower FVC and FEV1 values, higher FEV_1_/FCV ratios, reduced DLCO, higher GAP index scores and higher mMRC dyspnoea scores. No significant differences were observed in comorbidities and use of anti‐fibrotic agents between the two groups. Regarding CPET findings, the event group exhibited lower peak PETCO_2_, oxygen pulse, and peak workload, as well as higher O_2_ decrement, FAI, and $\overset{`}{V}$ E/$\overset{`}{V}$ CO_2_ slope.

**TABLE 2:** Comparison of clinical characteristics between survival and overall event.

	Survival	Events^a^	*p*
	(*n* = 148)	(*n* = 62)	
Age (years)	66 (58–74)	71 (66–76)	0.001**
Sex^b^			0.005**
Male	57 (38.5%)	37 (59.7%)	
Female	91 (61.5%)	25 (40.3%)	
Body mass index (kg/m^2^)	23.6 (21.4–26.0)	23.1 (20.6–26.7)	0.684
Classification of ILD^b^			< 0.001**
IPF	25 (16.9%)	31 (50.0%)	
CTD‐ILD	123 (83.1%)	31 (50.0%)	
Charlson comorbidity index	2 (1–4)	3 (2–5)	0.183
Comorbidity			
Cardiovascular diseases^b^	33 (21.9%)	15 (25.4%)	0.580
Cerebrovascular disease^c^	14 (9.3%)	6 (10.2%)	0.842
Pulmonary disease except ILD^b^	117 (77.5%)	50 (84.7%)	0.241
Chronic kidney disease^c^	29 (19.2%)	12 (20.3%)	0.852
Cancer^c^	17 (11.3%)	4 (6.8%)	0.331
Pulmonary function test			
FVC, % predicted	76.5 (63.3–88.8)	64.0 (48.8–80.0)	< 0.001**
FEV_1_, % predicted	78.0 (64.0–88.0)	67.0 (53.0–81.5)	0.007*
FEV_1_/FVC	82.0 (78.0–86.0)	86.0 (79.5–91.0)	0.012*
DLCO, % predicted	70.0 (54.0–84.5)	59.0 (40.5–71.5)	< 0.001**
GAP index	3 (1–4)	4 (3–6)	< 0.001**
mMRC dyspnoea score	1 (0–2)	2 (1–2)	< 0.001**
CPET data			
Gas exchange efficiency			
Peak PETCO_2_, mmHg	32.0 (28.1–35.8)	27.4 (22.5–30.8)	< 0.001**
O_2_ decrement	3 (2–6)	5 (2.3–8.8)	0.003**
Cardiovascular response			
Oxygen pulse, % predicted	84.0 (70.0–100.8)	67.0 (56.0–89.8)	< 0.001**
Heart rate recovery 1 min after CPET, beats/min	10 (5.3–15)	6 (2–10)	< 0.001**
Ventilation efficiency			
Breathing reserve, %	47.5 (39.0–58.0)	48.5 (29.8–57.8)	0.548
$\dot{V}E$/$\dot{V}{\mathrm{CO}}_{2}$ slope	31.8 (29.1–37.1)	38.2 (33.4–43.5)	< 0.001**
Summary			
FAI, %	33.0 (24.0–44.0)	46.2 (32.4–57.4)	< 0.001**
Peak workload	60.0 (40.0–77.5)	40.0 (30.0–60.0)	< 0.001**
Anti‐fibrotic agents during follow‐up^b^	72 (48.6%)	39 (62.9%)	0.059
Follow‐up time (years)	3.5 (2.2–4.9)	2.0 (1.0–3.1)	< 0.001**

*Note*: Data are presented as median (interquartile range) or *n* (%).

Abbreviations: CPET, cardiopulmonary exercise testing; CTD‐ILD, connective tissue disease‐associated interstitial lung disease; DLCO, diffusing capacity of the lungs for carbon monoxide; FAI, functional aerobic impairment; GAP, gender‐age physiology; IPF, idiopathic pulmonary fibrosis; mMRC, Modified Medical Research Council; PETCO_2_, end‐tidal carbon dioxide partial pressure; $\dot{V}\;E$/$\dot{V}\;{\mathrm{CO}}_{2}$, minute ventilation to carbon dioxide output; $\dot{V}\;O_{2}$, oxygen consumption.

^a^ Event: death or lung transplantation.

^b^ Chi‐Square test between IPF and CTD‐ILD.

^c^ Fisher's exact test between IPF and CTD‐ILD.

* *p* < 0.05.

** *p* < 0.01.

Cox regression analyses for event risk are summarised in Table 3. In the multivariable model, after adjusting for age, sex, ILD aetiology, and GAP index, FAI and $\overset{`}{V}$ E/$\overset{`}{V}$ CO_2_ slope emerged as positive predictors of events (HR = 1.03, *p* < 0.001; HR = 1.05, *p* < 0.001, respectively). In contrast, peak PETCO_2_, oxygen pulse, and heart rate recovery 1 min after CPET were negative predictors (HR = 0.93, *p* < 0.001; HR = 0.97, *p* < 0.001; HR = 0.95, *p* = 0.019, respectively). O_2_ decrement and breathing reserve were not significantly associated with event risk in the multivariable analysis (HR = 1.05, *p* = 0.062; HR = 0.99, *p* = 0.300, respectively).

**TABLE 3:** Cox regression analysis for cumulative overall event in interstitial lung disease.

	Simple model		Adjusted for age, sex, IPF and GAP index	
	HR (95% CI)	*p*	HR (95% CI)	*p*
Age (years)	1.03 (1.01–1.06)	0.004**		
Sex‐male (ref: female)	2.40 (1.44–4.00)	0.001**		
Classification‐IPF (ref: CTD‐ILD)	4.22 (2.52–7.04)	< 0.001**		
GAP index	1.47 (1.30–1.66)	< 0.001**		
CPET data				
Gas exchange efficiency				
Peak PETCO_2_, mmHg	0.90 (0.87–0.94)	< 0.001**	0.93 (0.89–0.97)	< 0.001**
O_2_ decrement	1.08 (1.03–1.13)	0.003**	1.05 (1.00–1.12)	0.062
Cardiovascular response				
Oxygen pulse, % predicted	0.98 (0.97–0.99)	< 0.001*	0.97 (0.96–0.99)	< 0.001**
Heart rate recovery 1 min after CPET, beats/min	0.93 (0.89–0.97)	0.001**	0.95 (0.90–0.99)	0.019*
Ventilation efficiency				
Breathing reserve, %	0.98 (0.997)	0.021*	0.99 (0.98–1.01)	0.300
$\dot{V}E$/$\dot{V}{\mathrm{CO}}_{2}$ slope	1.07 (1.05–1.10)	< 0.001**	1.05 (1.02–1.08)	0.001**
Summary				
FAI, %	1.03 (1.01–1.04)	< 0.001**	1.03 (1.01–1.05)	< 0.001**

*Note*: Cox regression.

Abbreviations: CPET, cardiopulmonary exercise testing; CTD‐ILD, connective tissue disease‐associated interstitial lung disease; FAI, functional aerobic impairment; IPF, idiopathic pulmonary fibrosis; PETCO_2_, end‐tidal carbon dioxide partial pressure; $\dot{V}E$/$\dot{V}{\mathrm{CO}}_{2}$, minute ventilation to carbon dioxide output; $\dot{V}O_{2}$, oxygen consumption.

** *p* < 0.01.

* *p* < 0.05.

### 3.3 Optimal Cutoff Value and Summed Score Development

**FIGURE 1:**
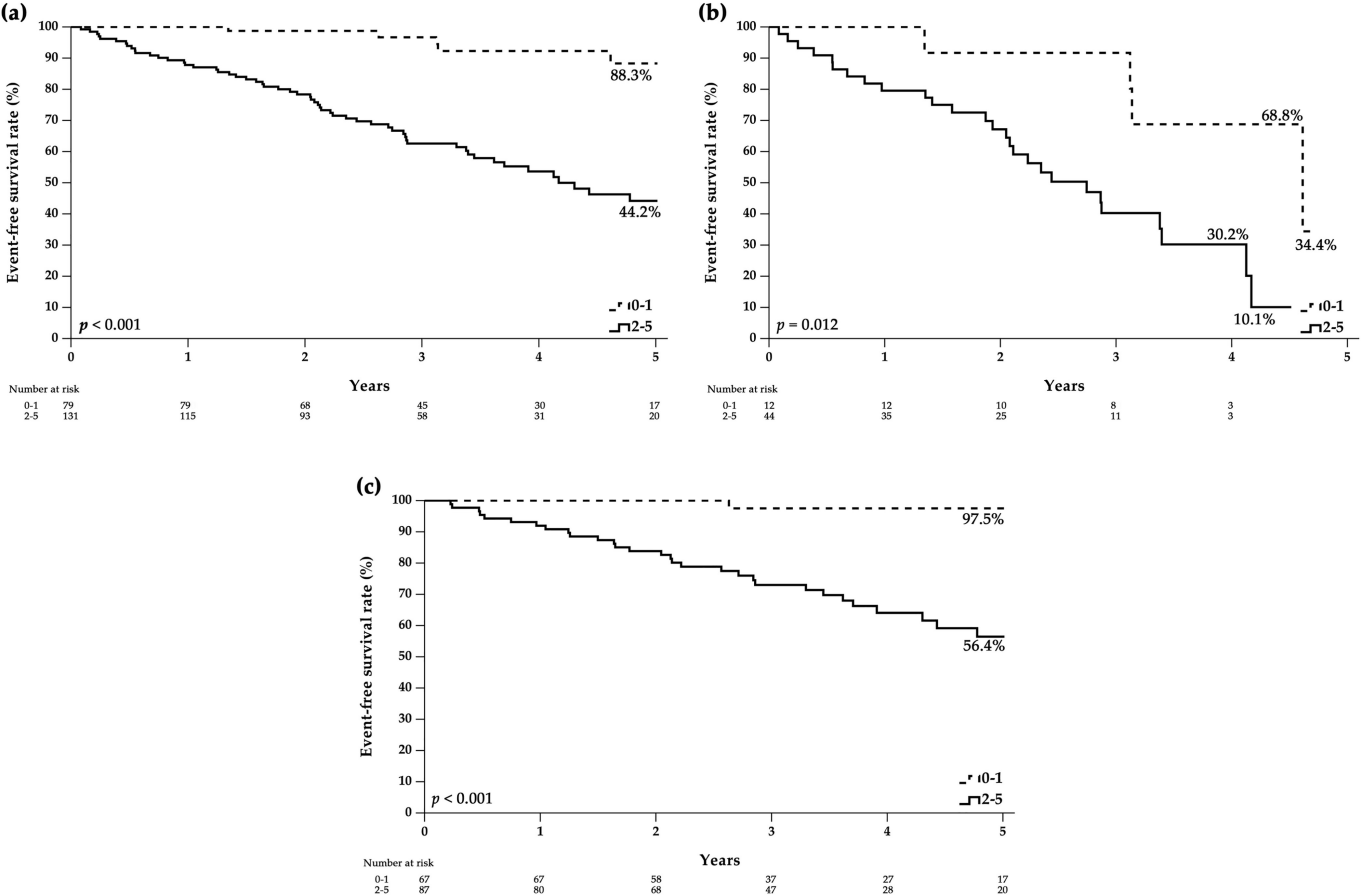
Kaplan–Meier event‐free survival analysis comparing summed scores of 0–1 versus 2–5 in subjects with (a) interstitial lung disease, (b) idiopathic pulmonary fibrosis, and (c) connective tissue disease‐associated interstitial lung disease.

Peak V̇O_2_ per body weight was excluded from the summed score due to its strong correlation with FAI and its relatively lower prognostic value in Cox regression analyses. O_2_ decrement and breathing reserve were also excluded because no significant difference was observed between the survival and event groups (*p* = 0.062 and *p* = 0.300, respectively). Finally, five CPET parameters (peak PETCO_2_, oxygen pulse, heart rate recovery 1 min after CPET, $\overset{`}{V}$ E/$\overset{`}{V}$ CO_2_, and FAI) was included in the summed score. As shown in Table 4, the newly developed 5‐point summed score—based on the number of CPET parameters exceeding their respective cutoff values—achieved the highest area under the curve (AUC) compared with any individual CPET parameter, with an optimal cutoff value of > 1. Kaplan–Meier event‐free survival curves for different summed score categories are presented in Figure 1. Patients with ILD who scored 2 to 5 had significantly lower event‐free survival rates during follow‐up compared to those who scored 0 to 1 (44.2% vs. 88.3%, *p* < 0.001).

**TABLE 4:** Possible predictors of overall events during follow‐up from cardiopulmonary exercise testing in subjects with interstitial lung disease.

CPET parameters	AUC (95% CI)	*p*	Cutoff point	Sensitivity	Specificity	PPV	NPV	Accuracy
Peak PETCO_2_, mmHg	0.747 (0.682–0.804)	< 0.001	≤ 31.24	80.7	59.2	45.5	87.9	65.6
$\dot{V}$E/$\dot{V}$CO_2_ slope	0.725 (0.659–0.784)	< 0.001	> 34	74.2	62.8	45.5	85.3	66.2
FAI, %	0.688 (0.621–0.750)	< 0.001	> 39.55	69.4	66.9	46.7	83.9	67.6
Oxygen pulse, % predicted	0.681 (0.613–0.743)	< 0.001	≤ 76	66.1	67.6	46.1	82.6	67.1
Heart rate recovery 1 min after CPET, beats/min	0.660 (0.591–0.723)	< 0.001	≤ 10	80.7	48.0	39.4	85.5	57.6
The summed score (1 point for each item above the cut point, maximal 5 points)^a^	0.785 (0.723–0.838)	< 0.001	> 1	91.9	50.0	43.5	93.7	62.4

Abbreviations: CPET, cardiopulmonary exercise testing; FAI, functional aerobic impairment; NPV, negative predictive value; PETCO_2_, end‐tidal carbon dioxide partial pressure; PPV, positive predictive value; $\dot{V}E$/$\dot{V}{\mathrm{CO}}_{2}$, minute ventilation to carbon dioxide output.

^a^ Five points included peak end‐tidal carbon dioxide partial pressure, slope of the minute ventilation‐to‐carbon dioxide output, functional aerobic impairment, oxygen pulse, and heart rate recovery 1 min after CPET.

### 3.4 Subgroup Analysis of Event Prediction Using the Summed Score in IPF and CTD‐ILD

**FIGURE 2:**
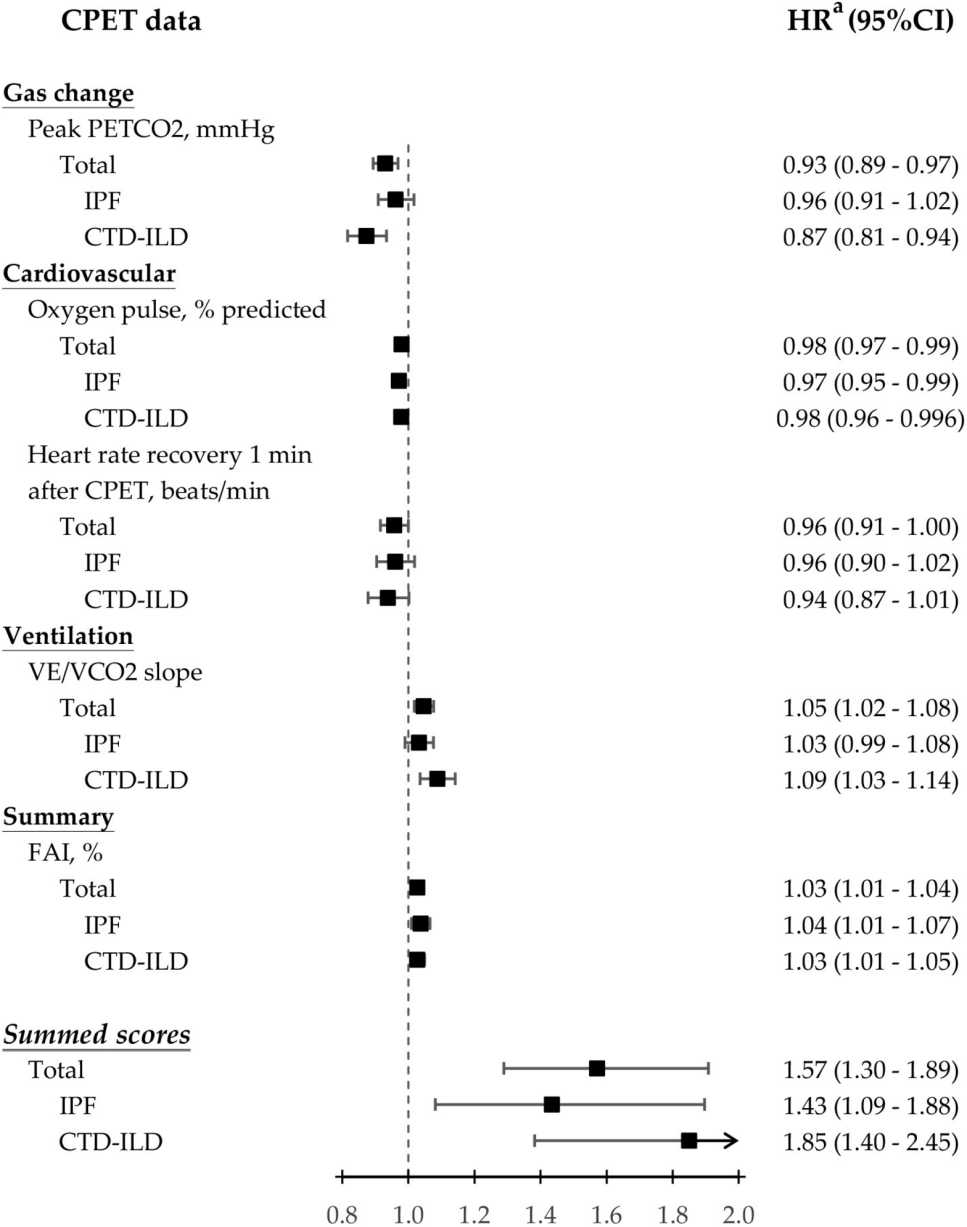
Forest plot of adjusted hazard ratios (HRa) for cardiopulmonary exercise testing parameters and summed score in predicting event risk during follow‐up of different subtypes of interstitial lung disease.

As shown in Figure 1, at the established cutoff value, the summed score demonstrated statistically significant differences in event‐free survival between scores of 2–5 and 0–1 for both IPF (*p* = 0.012) and CTD‐ILD (*p* < 0.001). However, in multivariable analyses adjusted for age, sex, and GAP index, peak PETCO_2_, heart rate recovery 1 min after CPET, and $\overset{`}{V}$ E/$\overset{`}{V}$ CO_2_ slope did not show significant predictive value for events in IPF. In addition, heart rate recovery 1 min after CPET also was not a significant predictor for events in CTD‐ILD. The summed score demonstrated significant predictive value for events in both IPF and CTD‐ILD. The detailed adjusted hazard ratios for mortality events are presented in Figure 2.

## 4 Discussion

In this study, we identified a summed score derived from five CPET parameters—peak PETCO_2_, FAI, oxygen pulse, heart rate recovery 1 min after CPET, and V̇E/V̇CO_2_ slope—as a significant predictor of mortality or the need for lung transplantation in newly diagnosed patients with IPF and CTD‐ILD. This work not only validates the CPET‐derived summed score as a prognostic tool for ILD but also evaluates its predictive performance across different ILD aetiologies, including IPF and CTD‐ILD. Although derived from a single‐centre cohort, to the best of our knowledge, this is the first prospective, multi‐year, real‐world study to assess the long‐term prognostic utility of this score.

Previous research using CPET parameters to predict outcomes in ILD patients is limited, and most available studies are small, retrospective, and single‐centre in design. A prospective observational study with a relatively small sample size (*n* = 45) in patients with systemic sclerosis–related ILD reported that a V̇E/V̇CO_2_ slope greater than 32 predicted 5‐year mortality [29]. Another single‐centre retrospective study in patients with advanced ILD found that peak workload and nadir SpO_2_ during CPET with supplemental oxygen were associated with mortality or the need for lung transplantation [30]. In contrast, the present study included a larger sample size, encompassed a broader range of ILD subtypes, and enrolled patients at an earlier disease stage, thereby enhancing the clinical relevance and generalisability of our findings.

CPET offers a comprehensive assessment of the respiratory, cardiovascular, and musculoskeletal responses to exercise under controlled laboratory conditions [26], with each parameter reflecting a distinct aspect of physical function. In this study, we systematically categorised CPET measures into four domains: gas exchange efficiency (peak PETCO_2_, O_2_ decrement), cardiovascular response (oxygen pulse, heart rate recovery 1 min after CPET), ventilatory efficiency (breathing reserve, V̇E/V̇CO_2_ slope), and overall CPET performance (FAI). Each parameter has specific prognostic value—for example, peak V̇O_2_ per body weight reflects maximal aerobic capacity and is linked to mortality risk in ILD [26], FAI provides an objective measure of cardiopulmonary impairment based on age‐ and sex‐predicted values, V̇E/V̇CO_2_ slope reflects gas exchange inefficiency and ventilation–perfusion mismatch [26], and oxygen pulse serves as an indirect estimate of stroke volume and is inversely associated with cardiovascular mortality risk [31]. While individual parameters may have limited predictive capacity when the AUC is modest, integrating multiple parameters into a summed score can enhance sensitivity, highlighting a key strength of CPET. This approach is consistent with the prognostic stratification framework jointly proposed by the European Association for Cardiovascular Prevention & Rehabilitation and the American Heart Association [32], which incorporates a composite scoring method similar to our 6‐point summed score. Notably, for the three overlapping parameters—PETCO_2_, heart rate recovery at 1 min, and V̇E/V̇CO_2_ slope—most of our cutoff values were classified outside the “green zone” defined in the guideline, reinforcing the consistency and validity of our findings. Taken together, these results support the potential use of CPET as a routine prognostic assessment tool for newly diagnosed ILD patients.

The results of this study are highly consistent with our previous findings [15], demonstrating a significant prognostic value of the CPET parameters for long‐term outcomes. Notably, the summed score remained a significant predictor of long‐term event when stratified by the IPF and CTD‐ILD subtypes, with survival curves showing statistically significant differences. Compared to our previous study, the event rate in this study was higher (29.5% vs. 7.5%), which reduces the risk of overfitting the results. The decrease in cutoff values for two parameters (FAI and $\dot{V}E$/$\dot{V}{\mathrm{CO}}_{2}$ slope) and the increase in cutoff values for the other three parameters (peak PETCO_2_, oxygen pulse and heart rate recovery 1 min after CPET) result in more subjects meeting the criteria to receive a score. In the previous study, the low‐summed score group consisted of 98 individuals, while the high‐summed score group included only 8, which represents only 7.4%. In contrast, the present study included 79 patients in the low‐summed score group and 131 in the high‐summed score group, representing 37.6% and 62.4% of the cohort, respectively. Furthermore, the previous study primarily enrolled patients with severely compromised physical function and assessed mortality over a 1‐year period, whereas this study extended the median follow‐up to 2.9 years and included a larger cohort with more favourable clinical profiles, thereby enhancing the generalizability and clinical applicability of the summed score.

Desaturation during exercise is a well‐recognised prognostic indicator in patients with ILD [26]. Alhamad et al. and Layton et al. also reported that the level of SpO_2_ below 85%–86% during CPET was significantly associated with increased mortality [30, 33]. However, the median SpO_2_ decrement in our subjects was only 4% (IQR: 2–7). This may be attributed to two factors. First, our patients were newly diagnosed, with mild disease severity based on lung function tests and mMRC dyspnoea scores. Second, most of the cohort consisted of CTD‐ILD cases, which are less progressive compared to IPF. When the O_2_ decrement cutoff was set at > 3%, the maximum AUC achieved was 0.63, which was lower than that of the other five CPET‐derived parameters. Regarding dead space, we did not include it in this study because we used a non‐invasive method that substitutes PETCO_2_ for arterial carbon dioxide pressure (PaCO_2_). This approach is known to underestimate the actual dead space in patients [34, 35].

This study has several limitations. First, it was a single‐centre cohort with limited generalisability and a relatively small overall sample size, particularly for the IPF subgroup. Second, this study included only IPF and CTD‐ILD, and other ILD subtypes were not assessed. Given the heterogeneity of ILD—with IPF typically progressing faster than CTD‐ILD [18], and CTD‐ILD prognosis varying by the underlying connective tissue disease—future studies should include subgroup analyses of specific CTD‐ILD types. Third, medical therapies, including anti‐fibrotic agents administered during follow‐up, may have introduced confounding effects. In Taiwan, anti‐fibrotic agents are subject to specific reimbursement criteria; many patients receiving anti‐fibrotic agents did so at their own expense because their lung function was too impaired to meet national health insurance coverage requirements. Consequently, the higher mortality observed in these patients may reflect the severity of their underlying disease rather than the effect of the medication itself—an inherent and unavoidable source of bias in real‐world studies. Fourth, although the thresholds were derived using an empirical, censor‐unadjusted ROC approach that may misclassify survival status, the summed score still showed prognostic value in the derivation cohort, supporting its robustness despite methodological limitations. Fifth, the CPET‐based model is not applicable to patients unable to complete CPET, such as those with significant musculoskeletal limitations or unstable disease activity. Another limitation is that in patients with CTD‐ILD, chronic musculoskeletal manifestations (such as arthritis, myositis, or general deconditioning) may restrict exercise performance independent of pulmonary involvement.

Future large, well‐controlled prospective studies are needed to validate the association between the summed score and mortality across diverse ILD subtypes and to minimise bias. Although CPET is not yet a routine tool in ILD assessment, our findings underscore its prognostic value in newly diagnosed patients and support broader clinical adoption, similar to its established role in pulmonary hypertension evaluation [36].

In conclusion, in this prospective observational cohort study, our findings highlight the prognostic value of a CPET‐derived summed score in predicting event‐free survival over a median follow‐up of 2.9 years and support its integration into MDD for the management of IPF and CTD‐ILD. Further studies are warranted to validate its applicability across other ILD subtypes.

## Author Contributions

**Yu‐Lin Tsai:** conceptualization (equal), funding acquisition (equal), methodology (equal), writing – original draft (lead). **Kai‐Ming Chang:** data curation (equal), investigation (equal), resources (equal), supervision (equal). **Chung‐Shih Chin:** data curation (equal), investigation (equal), supervision (equal). **Chiann‐Yi Hsu:** formal analysis (equal), methodology (equal), software (equal), visualization (equal). **Yi‐Hsuan Yu:** data curation (lead). **Yuan‐Yang Cheng:** conceptualization (equal), data curation (equal), project administration (equal), supervision (equal), writing – review and editing (equal). **Pin‐Kuei Fu:** conceptualization (equal), methodology (equal), project administration (equal), writing – review and editing (equal).

## Funding

The authors gratefully acknowledge partial funding support from the Department of Medical Research of Taichung Veterans General Hospital (TCVGH‐1137308C; TCVGH‐1123104D; TCVGH‐1128303D; TCVGH‐1137308D; TCVGH‐1136601A) and the National Science and Technology Council of Taiwan (NSTC 112‐2314‐B‐075A‐003‐MY3), which covered study manpower, materials, and publication fees. The funding source was not involved in the study's design, data collection, data analysis, interpretation of results, or the preparation of this manuscript.

## Ethics Statement

The study was conducted in accordance with the Declaration of Helsinki and approved by the Institutional Review Board of Taichung Veterans General Hospital (Approval No. CG24056C). Written informed consent was obtained from all participants. This study is a subgroup analysis of the Registry of Interstitial Lung Disease (REGILD), a prospective multicentre ILD registry in Taiwan (NCT06476470) https://clinicaltrials.gov/study/NCT06476470?cond=NCT06476470&rank=1.

## Conflicts of Interest

The authors declare no conflicts of interest.

## Data Availability

The datasets generated and analysed during the current study are available through the corresponding author upon reasonable request.
